# Association of Resting Heart Rate and Heart Rate Variability With Proximal Suicidal Risk in Patients With Diverse Psychiatric Diagnoses

**DOI:** 10.3389/fpsyt.2021.652340

**Published:** 2021-04-30

**Authors:** Dongbin Lee, Ji Hyun Baek, Yun Ji Cho, Kyung Sue Hong

**Affiliations:** Samsung Medical Center, Sungkyunkwan University School of Medicine, Seoul, South Korea

**Keywords:** suicide, resting heart rate, resting heart rate variability, root-mean-square of successive difference, biomarker

## Abstract

Objectively measurable biomarkers have not been applied for suicide risk prediction. Resting heart rate (HR) and heart rate variability (HRV) showed potential as trans-diagnostic markers associated with suicide. This study aimed to investigate the associations of resting HR and HRV on proximal suicide risk in patients with diverse psychiatric diagnoses. This chart review study used the medical records of psychiatric patients who visited the outpatient clinic at an academic tertiary hospital. A total of 1,461 patients with diverse psychiatric diagnoses was included in the analysis. Proximal suicide risk was measured using the Mini-International Neuropsychiatric Interview (MINI) suicidal score. Linear regression analyses with the MINI suicidal score as a dependent variable and binary logistic regression analyses with moderate-to-high suicide risk (MINI suicidal risk score ≥6) as a dependent variable were conducted to explore the effects of resting HR and HRV parameters on acute suicide risk after adjusting for age, sex, presence of major depressive disorder (MDD) and bipolar disorder (BD), severity of depression and anxiety severity. We found that 55 (34.6%) patients in the MDD group, 40 (41.7%) in the BD group and 36 (3.9%) in the others group reported moderate-to-high suicide risk. Linear regression analysis revealed that both resting HR and root-mean-square of successive difference (RMSSD) had significant associations with the MINI suicidal score (*P* = 0.037 with HR, *P* = 0.003 with RMSSD). In logistic regression, only RMSSD showed a significant association with moderate-to-high suicide risk (*P* = 0.098 with HR, *P* = 0.019 with RMSSD), which remained significant in subgroup analysis with patients who reported any suicide-related symptom (MINI suicidal score >0; *n* = 472; *P* = 0.017 with HR, *P* = 0.012 with RMSSD). Our study findings suggest the potential for resting HR and RMSSD as biomarkers for proximal suicide risk prediction. Further research with longitudinal evaluation is needed to confirm our study findings.

## Introduction

Suicide is a significant mental health problem. Extensive efforts have been made toward suicide risk prediction and prevention. In clinical practice, suicide risk assessment generally is based on clinical observation of the patient's current state and previous history. However, the validity, reliability, and utility of current suicide risk prediction systems are unknown (1). To increase the accuracy of suicidal risk prediction, objectively measurable biomarkers that can discriminate patients in an “at-risk” state are needed.

Physiological biomarkers that can be measured when an individual is in an “at-risk” state are promising candidates for suicide risk monitoring. Heart rate (HR) and heart rate variability (HRV) can be used for this purpose. HRV refers beat-to-beat variations in HR. Both HR and HRV are produced by sympathetic and parasympathetic neural activity. Lower HRV is an indicator of dysregulation of cardiac autonomic function and a predictor of poor health status (2). Short-term power spectral analysis of HRV, which has been standardized since 1996 (3), has been developed as a reliable and non-invasive tool to probe the autonomic regulation of the heart. Several studies have reported that resting HR (4, 5) and resting HRV (6) showed associations with suicide attempts.

Despite the potential of resting HR and resting HRV as candidate biomarkers for “at risk” states, previous studies have limitations with respect to generalizing their results. First, aside from limited sample sizes, most studies have explored associations with suicide ideation (7–9) or lifetime suicide attempts (9, 10). To assess the potential of resting state HR and HRV as biomarkers to identify the “at-risk” state, an association with “proximal” suicide risk should be examined. Second, most studies included biased populations in terms of psychiatric diagnoses. Suicide is a complex phenomenon that can occur among individuals suffering from a range of diverse psychiatric conditions (11). Focusing on populations with certain psychiatric disorders can hinder generalization of study findings. In particular, patients with subthreshold disorder manifestations have not been included in previous studies. Considering the dimensional nature of suicidality, patients with subthreshold disorder manifestations that can involve significant distress and dysfunction (12) should be included in studies exploring potential biomarkers. In addition, resting HRV is a biomarker for various psychiatric conditions. Meta-analytic studies have shown that decreased HRV is associated with diverse psychiatric illnesses including major depressive disorder (MDD) (13), bipolar disorder (BD) (14), anxiety disorder (15), and schizophrenia (16), indicating a broad association of decreased HRV with psychopathology rather than with a single disorder. To determine the association between resting HRV and suicide risk, additional investigations that include diverse psychiatric populations are needed.

In the present study, we examined the association of resting HR and HRV with proximal suicide risk in patients with diverse psychiatric diagnoses. Using a large clinical population with diverse psychiatric conditions including subthreshold disorder manifestations, we investigated the associations of resting HR and HRV with proximal suicide risk.

## Methods

### Study Population

Data were obtained from retrospective chart reviews of patients who visited the outpatient clinic of Samsung Medical Center between January 1, 2017 and December 31, 2019. As part of routine care, all patients who visited the outpatient clinic of the Department of Psychiatry at Samsung Medical Center underwent a standardized evaluation process including psychological and biological evaluations. Following an initial interview with a board-certified psychiatrist, patients between 18 and 65 years of age were referred for the evaluation process. The psychological evaluation was designed to confirm the clinical diagnosis of subjects based on the *Diagnostic and Statistical Manual of Mental Disorders*, fourth edition, text revision (DSM-IV-TR), and to evaluate symptom severity. The psychologist who conducted the evaluation had more than 2 years of clinical experience. Detailed psychological measures included in the evaluation process were described elsewhere (17). Patients suspected of psychosis or intellectual disability or those who required emergency admission were not referred for the evaluation. Because this study targeted trans-diagnostic features associated with emotional disorder, we also excluded any subject who were suspected to have schizophrenia and related disorders. In this study, we analyzed the test results of comprehensive psychological evaluations and resting HRV measures. A total of 1,461 subjects was included in the analysis.

The study protocol was approved by the Institutional Review Board of Samsung Medical Center (no. 2018-11-019). The study was performed in accordance with the ethical standards stated in the 1964 Declaration of Helsinki. All identifying data were removed from the clinical database prior to analyses. The need for patient consent was waived because this was a retrospective chart review study.

### Applied Measures

#### Psychiatric Diagnoses

The diagnoses of study subjects were evaluated using the Korean version of the Mini International Neuropsychiatric Interview (MINI) (18). MINI diagnostic modules for major depressive disorder, dysthymia, manic episode, panic disorder, agoraphobia, social phobia, obsessive-compulsive disorder, posttraumatic stress disorder, alcohol dependence, schizophrenia, delusional disorder, generalized anxiety disorder, somatoform disorder, adjustment disorders and attention deficit hyperactivity disorder were included. When symptoms did not meet the full criteria, diagnoses with not otherwise specified were coded. Primary psychiatric diagnoses based on the DSM-IV-TR (19) diagnoses were included for the analysis.

#### Emotional Symptoms

The Hamilton rating scale for depression (HAM-D) (20) was adopted to evaluate current depressive symptoms in patients. The Hamilton rating scale for anxiety (HAM-A) (21) was used to evaluate anxiety symptoms.

#### Proximal Suicide Risk

The proximal suicide risk was measured using the MINI suicide-item modules. The MINI suicide module is a six-question, yes-or-no, interviewer-administered questionnaire used to evaluate suicidality. The MINI suicide module assesses experiences during the past month of recurrent thoughts of death (1 point), ideas of self-harm (2 points), presence of suicide ideation (6 points), plans for suicide (10 points), and suicide attempts (10 points). In addition, lifetime history of suicide attempts (4 points) is assessed. The MINI suicidal score is calculated by adding the assigned points of the items checked as “yes” in the suicide module. We used the MINI suicidal score as a marker for proximal suicide risk. A previous study that examined the predictive validity of the MINI suicidal scale reported good sensitivity (0.61–0.75) and specificity (0.61–0.75) in predicting suicidal behavior. The suicide risk score is categorized as low risk (<6 points), moderate risk (6–9 points), or high risk (≥10 points) for future suicide attempts (22).

#### Resting HRV

Data collection and HRV analysis were completed using SA-6000 (Medicore Co., Seoul, Korea). HRV data analysis and signal processing followed guidelines defined by the Task Force of the European Society of Cardiology and the North American Society of Pacing and Electrophysiology (23). The measurement was conducted between 9 A.M. and 4 P.M. Prior to undergoing measurement, patients were instructed to refrain from intake of alcohol, caffeine, and food and from smoking for at least 2 h before the test. After 5 min of rest, an electrocardiogram was recorded for 3 min with the patient in a sitting position. We calculated HR and three features based on R-R interval using time-domain analysis, standard deviation of average normal-normal intervals (SDNN), and root mean square of successive differences (RMSSD). The SDNN reflects both sympathetic and parasympathetic activities, while the RMSSD is sensitive to parasympathetic modulation (24). Frequency domain features indicate various spectral components, including low frequency (LF: 0.04~0.15 Hz), high frequency (HF: 0.15~0.4 Hz) and the LF/HF ratio. LF is modulated by sympathetic and parasympathetic activities, and HF is modulated by parasympathetic activities. The LF/HF ratio measures the balance between sympathetic and parasympathetic activities (25).

### Statistical Analyses

We divided participants into three groups based on psychiatric diagnoses associated with high suicide risk: MDD, BD, and others. We compared demographic characteristics, depression and anxiety severity, frequency of each item of the MINI suicide module, resting HR, and HRV measures among the three groups. Analyses of variances (ANOVA) were used for continuous variables, and chi-square test was used for categorical variables. *Post-hoc* analyses were conducted using the Fisher's Least Significant Difference (LSD) method. We performed multivariate linear regression analyses to determine associations of resting-state HR and HRV measures with proximal suicide risk. The MINI suicidal score was entered as a dependent variable in linear regression analyses. Age, sex, diagnostic group (MDD, BD, and others), depression, and anxiety severity were entered as covariates. As most previous studies focused on suicidality in mood disorders, MDD and BD also were entered as covariates. To examine whether resting HR and HRV measures could differentiate patients with moderate-to-high suicide risk among patients who reported any kind of suicide-related symptom, multivariate logistic regression analysis was performed in a subgroup of patients with MINI suicidal score >0. The significance level was set at *P* < 0.05. All statistical analyses were performed using R version 4.02 (26).

## Results

A total of 1,461 patients (mean age of 42.05 years, 36.8% male) was included in the study analysis. Table 1 lists the primary psychiatric diagnoses of the patients as determined by the MINI. Among the total 1,461 patients, 448 (30.7%) had MDD, 222 (15.2%) had panic disorder, 148 (10.1%) had anxiety disorder other than panic disorder, 102 (7.0%) had somatic symptom or related disorder, and 96 (6.6%) had bipolar disorder. Seventy-three (5.0%) patients did not experience psychiatric symptoms that met the DSM criteria for any disorder and were assigned as having a subthreshold diagnosis.

**Table 1:** Primary diagnoses of patients included in the analysis.

**Diagnosis**	***n* (%)**
Major depressive disorder	448 (30.6)
Bipolar and related disorder	96 (6.6)
Others	917 (62.8)
Attention deficit/hyperactivity disorder	9 (0.6)
Adjustment disorder	126 (8.6)
Alcohol use disorder	16 (1.1)
Anxiety disorder	148 (10.1)
Depressive disorder other than major depressive disorder	124 (8.5)
Insomnia disorder	30 (2.1)
Obsessive-compulsive and related disorder	16 (1.1)
Panic disorder	222 (15.2)
Posttraumatic stress disorder and acute stress disorder	22 (1.5)
Somatic symptom and related disorder	102 (7.0)
Tic disorder	13 (0.9)
Others	16 (1.1)
Subthreshold diagnoses	73 (5.0)
Total	1,461 (100)

*MDD, major depressive disorder*.

**Figure 1:**
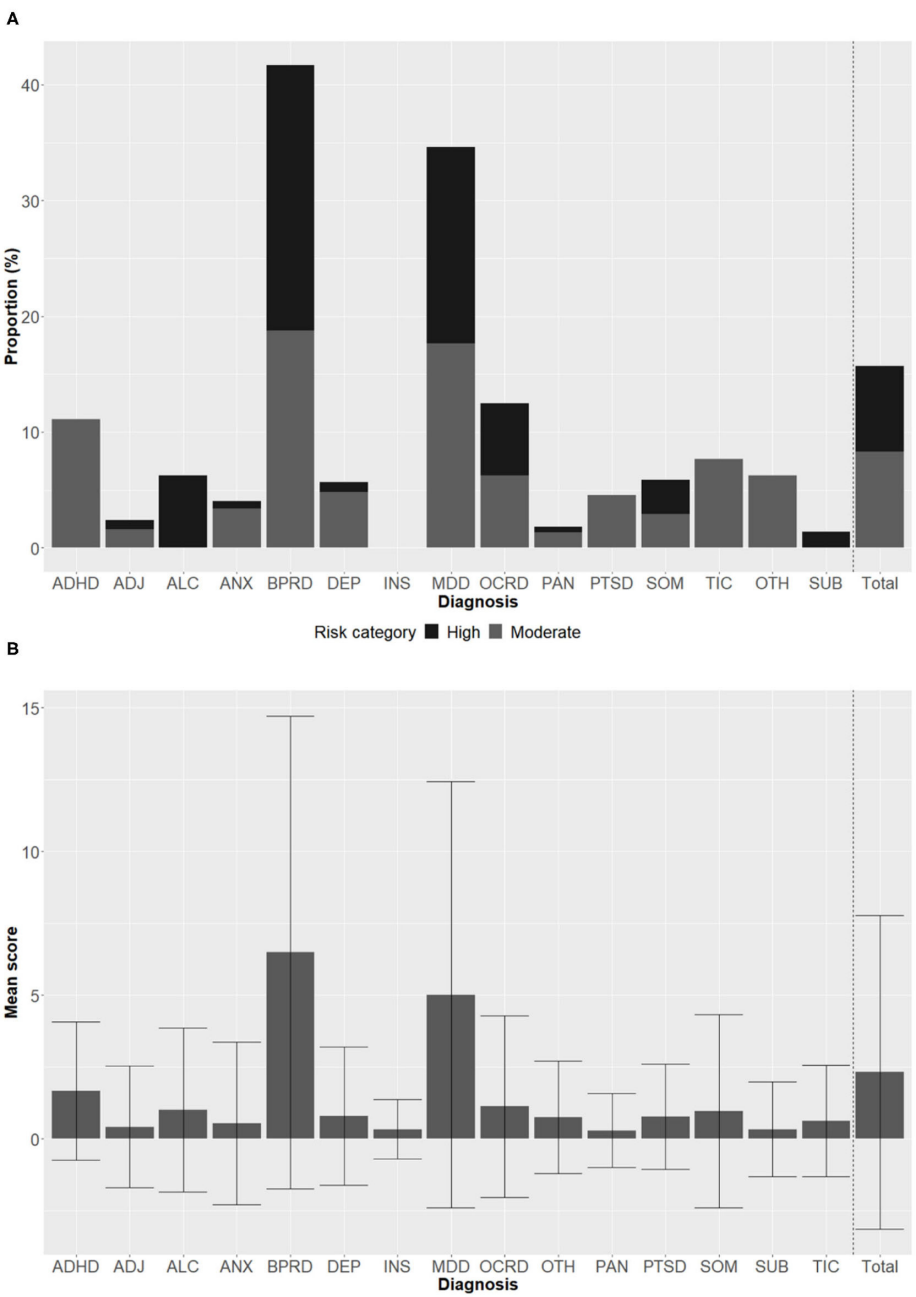
Distribution of MINI suicidal scores across primary diagnoses. MINI suicidal score was categorized as low risk (<6 points), moderate risk (6–9 points), or high risk (≥10 points). Proportions of subjects with moderate risk and high risk in each primary diagnosis **(A)**. Mean MINI suicidal score in each primary diagnosis **(B)**. Error bars represent standard deviation. MINI, Mini-International Neuropsychiatric Interview; ADHD, attention deficit hyperactivity disorder; ADJ, adjustment disorder; ALC, alcohol use disorder; ANX, anxiety disorder other than panic disorder; BPRD, bipolar and related disorders; DEP, depressive disorder other than major depressive disorder; INS, insomnia disorders; MDD, major depressive disorder; OCRD, obsessive-compulsive and related disorders; PAN, panic disorder; PTSD, posttraumatic stress disorder or acute stress disorder; SOM, somatic symptom and related disorders; TIC, tic disorders; OTH, others; SUB, subthreshold diagnosis.

Among the total 1,461 patients, 472 (32.3%) responded “yes” to at least one item of the MINI suicide module (MINI suicidal score >0), while 229 (15.7%) showed moderate-to-high risk based on the MINI suicidal score. The primary psychiatric diagnoses of patients were diverse (Figure 1). The mean MINI suicidal score was highest in the BD group [6.49 standard deviation (SD) = 8.22)], followed by the MDD group [5.01 (SD = 7.42); Table 2]. Likewise, patients with moderate-to-high suicidal risk were most common in BD (41.7%) and MDD (34.6%) groups. Of patients with non-MDD, 19.2% (*n* = 194) responded “yes” to at least one item of the MINI suicide module, and 7.3% (*n* = 74) had moderate-to-high suicide risk.

**Table 2:** Comparisons of demographic, clinical characteristics and suicidal risk between major depressive disorder and other diagnoses.

	**MDD^**1**^** **(*n* = 448)**	**BD^**2**^** **(*n* = 96)**	**Others^**3**^** **(*n* = 917)**	***X^**2**^* or *F***	***P***	***Post-hoc*** **analysis**		
						***^**1**^ vs. ^**2**^***	***^**2**^ vs. ^**3**^***	***^**3**^ vs. ^**1**^***
**Demographic and clinical variables**								
Age, mean (SD)	41.11 (13.85)	32.20 (10.51)	43.52 (13.04)	33.87	<0.001	<0.001	<0.001	0.001
Sex, male	125 (27.9)	46 (47.9)	368 (40.1)	24.50	<0.001	<0.003	0.405	<0.003
HAM-D score, mean (SD)	20.01 (6.01)	17.30 (7.19)	13.16 (5.90)	198.72	<0.001	<0.001	<0.001	<0.001
HAM-A score, mean (SD)	20.65 (6.34)	17.76 (7.88)	16.44 (6.92)	57.04	<0.001	<0.001	<0.001	0.071
**MINI suicide module**								
Recurrent thought of death, n (%)	257 (57.4)	55 (57.3)	116 (12.6)	33,036	<0.001	1.000	<0.003	<0.003
Idea of self-harm, n (%)	34 (7.6)	13 (13.5)	9 (1.0)	62.08	<0.001	0.180	<0.003	<0.003
Suicidal idea, n (%)	153 (34.2)	38 (39.6)	33 (3.6)	263.62	<0.001	1.000	<0.003	<0.003
Suicide plan, n (%)	39 (8.7)	12 (12.5)	5 (0.5)	75.48	<0.001	1.000	<0.003	<0.003
Recent suicide attempt, n (%)	26 (5.8)	7 (7.3)	3 (0.3)	FE	<0.001	1.000	<0.003	<0.003
Lifetime suicide attempt, n (%)	88 (19.6)	31 (32.3)	30 (3.3)	62.73	<0.001	0.021	<0.003	<0.003
MINI suicidal score, mean (SD)	5.01 (7.42)	6.49 (8.22)	0.58 (2.33)	155.8	<0.001	0.008	<0.001	<0.001

*MDD, major depressive disorder; BD, bipolar disorder; HAM-D, Hamilton depression scale; HAM-A, Hamilton anxiety scale; MINI, mini international neuropsychiatric interview*.

In terms of demographic characteristics, females were more common in the MDD group. The BD group was youngest, followed by the MDD group and others. Depression and anxiety severity, all items in the MINI suicidal module, and MINI suicidal score were highest in the BD group, followed by the MDD group and others.

Resting state HR and HRV measures were compared among the three groups (Table 3). Significant differences were observed in resting HR, SDNN, and LF between groups. In *post-hoc* analyses, the MDD group had lower resting HR, SDNN, and LF compared with the BD group and a lower SDNN compared with the others group.

**Table 3:** Comparison of HRV measures between MDD, BD, and others group.

	**MDD^**1**^** **(*n* = 448)**	**BD^**2**^** **(*n* = 96)**	**Others^**3**^** **(*n* = 917)**	***F***	***P***	***Post-hoc*** **analysis**		
						***^**1**^ vs. ^**2**^***	***^**2**^ vs. ^**3**^***	***^**3**^ vs. ^**1**^***
HR (bpm)	79.42 (13.12)	80.00 (12.88)	76.86 (12.24)	7.76	<0.001	0.679	0.549	0.386
RMSSD (ms)	20.73 (12.21)	23.94 (13.24)	21.42 (11.82)	−1.36	0.174			
SDNN (ms)	28.72 (13.29)	32.36 (13.83)	30.65 (13.93)	4.31	0.014	0.018	0.246	0.014
LF (ms^2^/Hz)	234.82 (367.47)	366.63 (631.26)	274.48 (526.75)	3.031	0.049	0.017	0.081	0.162
HF (ms^2^/Hz)	155.92 (197.06)	200.59 (237.62)	174.12 (244.50)	1.83	0.161			
LF/HF ratio	2.46 (3.22)	3.03 (4.69)	2.71 (5.68)	0.67	0.513			

*MDD, major depressive disorder; BD, bipolar disorders; HR, resting state heart rate; RMSSD, root mean square of R-R interval of successive differences; SDNN, standard deviation of average normal-normal intervals; LF, low frequency (0.04~0.15 Hz); HF, high frequency (0.15~0.4 Hz)*.

**Figure 2:**
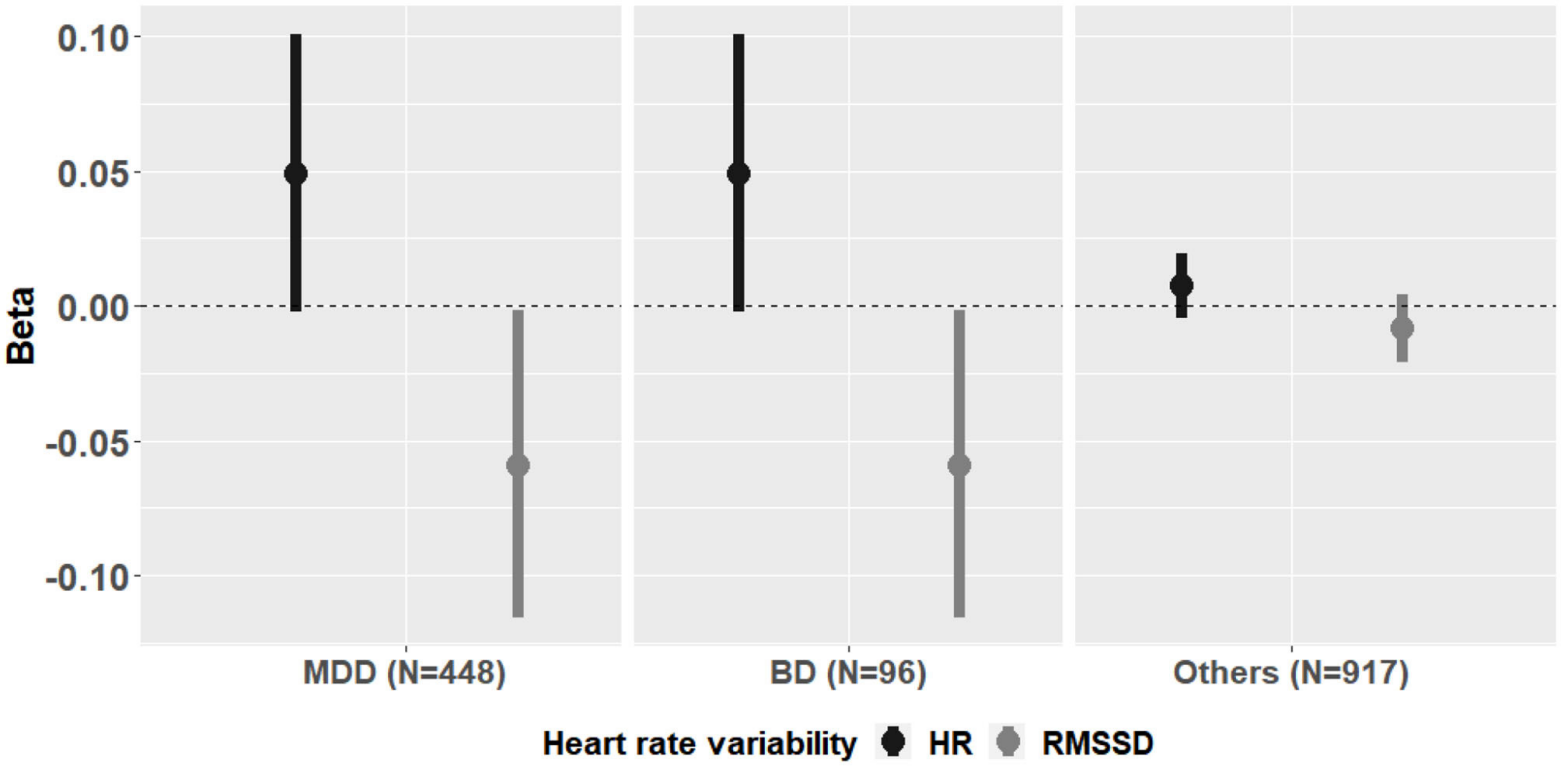
Association between heart rate variability and MINI suicidal scale in two subgroups of major depressive disorder and other diagnoses. MINI, Mini-International Neuropsychiatric Interview; MDD, major depressive disorder; RMSSD, root mean square of R-R interval of successive differences; HR, resting state heart rate.

In linear regression analyses, both HR (*P* = 0.037) and RMSSD (*P* = 0.003) showed significant association with the MINI suicidal score after adjusting for age, sex, primary diagnosis, and HAM-D and HAM-A scores (Table 4). The overall directions of the association were similar across MDD, BD and others groups (Figure 2). Other HRV measures did not show significant associations with the MINI suicidal score. In logistic regression analyses, only RMSSD showed a significant association with moderate-to-high suicide risk after adjusting for the aforementioned covariates (*P* = 0.098 with HR, *P* = 0.019 with RMSSD; Table 5). When we only included participants with any suicide-related symptom (MINI suicidal score >0), a total of 472 subjects with MINI suicidal score 0 was included in the analyses. Both HR (*P* = 0.010) and RMSSD (*P* = 0.002) showed significant associations with MINI suicidal score. In logistic regression analyses, HR (*P* = 0.017) and RMSSD (*P* = 0.012) showed significant associations with moderate-to-high suicide risk.

**Table 4:** Multivariate linear regression analysis of association of resting heart rate (HR) and root-mean-square of R-R interval successive difference with the MINI suicidal scale.

	**Unstandardized beta**	**SE**	**Standardized beta**	***t***	***P***
**Model 1: HR as an independent variable**					
HR	0.021	0.010	0.049	2.085	0.037
Age	−0.037	0.010	−0.090	−3.742	<0.001
Sex	0.431	0.264	0.038	1.634	0.102
HAM-D score	0.291	0.032	0.361	9.167	<0.001
HAM-A score	−0.063	0.028	−0.082	−2.254	0.024
Primary diagnosis, MDD	2.621	0.312	0.220	8.388	<0.001
Primary diagnosis, bipolar disorder	4.267	0.529	0.193	8.065	<0.001
**Model 2: RMSSD as an independent variable**					
RMSSD	−0.032	0.011	−0.070	−2.968	0.003
Age	−0.049	0.010	−0.120	−4.910	<0.001
Sex	0.392	0.264	0.0.35	1.486	0.138
HAM-D score	0.293	0.032	0.364	9.288	<0.001
HAM-A score	0.066	0.028	−0.085	−2.342	0.019
Primary diagnosis, major depressive disorder	2.611	0.312	0.220	8.367	<0.001
Primary diagnosis, bipolar disorder	4.271	0.528	0.194	8.086	<0.001

*MINI, Mini-International Neuropsychiatric Interview; HR, resting state heart rate; SE, standard error; HAM-D, Hamilton depression scale; HAM-A, Hamilton anxiety scale; RMSSD, root mean square of R-R interval of successive differences*.

**Table 5:** Multivariate logistic regression analysis of association of resting heart rate (HR) and root-mean-square of R-R interval successive difference with the moderate-to-high suicide risk.

	**aOR**	**95% CI**	***P***
**Model 1: HR as an independent variable**			
HR	1.012	0.998–1.026	0.098
Age	0.969	0.956–0.983	<0.001
Sex	0.844	0.582–1.225	0.372
HAM-D score	1.239	1.185–1.296	<0.001
HAM-A score	0.950	0.914–0.987	0.008
Primary diagnosis			<0.001
- Major depressive disorder	5.237	3.428–8.001	<0.001
- Bipolar and related disorder	8.037	4.436–14.563	<0.001
**Model 2: RMSSD as an independent variable**			
RMSSD	0.981	0.966–0.997	0.019
Age	0.962	0.948–0.976	<0.001
Sex	0.871	0.599–1.267	0.460
HAM-D score	1.241	1.186–1.298	<0.001
HAM-A score	0.949	0.913–0.986	0.008
Primary diagnosis			<0.001
- Major depressive disorder	5.237	3.427–8.005	<0.001
- Bipolar and related disorder	8.142	4.487–14.774	<0.001

*MINI, Mini-International Neuropsychiatric Interview; HR, resting state heart rate; SE, standard error; HAM-D, Hamilton depression scale; HAM-A, Hamilton anxiety scale; RMSSD, root mean square of R-R interval of successive differences*.

Of all patients included in the analyses, 738 (50.0%) completed the alcohol use disorders identification test (AUDIT) (27). An AUDIT score ≥8 suggests harmful alcohol consumption. When we adjust harmful alcohol consumption in regression analyses to determine the effects of HR and HRV on suicide risk, resting HR and RMSSD showed significant association with the MINI suicidal score in linear regression analyses (Standardized beta = 0.074, *t* = 2.23, *P* = 0.026 for HR; standardized beta = 0.070, *t* = −2.073, *P* = 0.039 for RMSSD), while none of them show significant associations with the moderate-to-high suicide risk in logistic regression analysis (*P* = 0.170 for HR; *P* = 0.167 for RMSSD).

## Discussion

Suicide is a major mental health problem that remains difficult to prevent. Finding objectively measurable biomarkers to detect proximal suicide risk could change the current approaches to suicide prevention. In this study, we examined whether resting-state HR and HRV measures showed significant associations with proximal suicide risk in patients with diverse psychiatric conditions. We found that resting-state HR and RMSSD were associated with proximal suicide risk, suggesting their potential as biomarkers for suicide risk prediction.

In our study, we included patients with diverse psychiatric conditions. The MDD group had more females compared with other diagnostic groups, which is consistent with previous findings that MDD is more commonly observed in females (28). Patients with diverse psychiatric diagnoses reported suicide-related symptoms. Approximately 4% of individuals in the others group showed a moderate-to-high suicide risk. A recent review suggested that a person with suicidal thoughts is at risk even if there are few overt symptoms of psychiatric diagnoses (29). Considering that suicidal behavior was observed trans-diagnostically, suicidal behavior disorder was suggested as a different psychiatric diagnostic entity in the DSM-5 (30).

Most previous studies on resting HRV focused on comparisons between patients with a single psychiatric disorder and healthy controls. Meta-analytic studies have shown that decreased HRV is associated with diverse psychiatric illnesses including MDD (13), BD (14), anxiety disorder (15), and schizophrenia (16), indicating an association of decreased HRV with psychopathology in general rather than with a single disorder. Whether there are diagnosis-specific differences in resting HRV measures remains unclear. Other factors including symptom severity could affect the differences between diagnostic groups. In our study, the overall directions of association were similar across diverse diagnostic groups (Figure 2), and the results remained significant even before adjusting for diagnostic groups.

Previous studies showed that a heightened autonomic state measured using HR and HRV (i.e., higher HR and lower variance of RR intervals) were associated with suicide (6). However, limited research has explored the associations of HR and HRV with proximal suicide risk. Most studies focused on lifetime suicide attempt or suicide ideation, which cannot be used to evaluate if an individual is an “at-risk” state. In our study, resting-state HR and RMSSD both showed significant associations with proximal suicide risk measured using the MINI suicidal score. In addition, the association remained significant in subgroup analysis with patients who reported any kind of suicide- related symptoms only (patients with a MINI suicidal score >0), indicating the potential of resting HR and RMSSD as a biomarker used for proximal suicide risk prediction.

Both HR and HRV are controlled by the autonomic nervous system. In particular, RMSSD is a marker of resting vagal tone. Cortical activity modulates cardiac activity via the vagus nerve (31). In addition, the orbitofrontal cortex (OFC) and medial prefrontal cortex (mPFC) tonically inhibit the amygdala via the vagal pathway (32). Thus, reduced vagal tone could reflect the activity of OFC and mPFC, which are associated with cognitive control in relation to suicide attempt (33). Prior studies have demonstrated the association between vagally-mediated resting state HRV and cognitive (34) and affective flexibility (35). Cognitive inflexibility can lead to suicidal behavior during a crisis, in which patients consider suicide the only way to escape their current distress (36).

In the Research Domain Criteria (RDoC) project that aims to identify trans-diagnostic dimensions of psychiatric illnesses, resting HR and HRV are affected by the arousal and regulatory system. This system is one of the least studied areas of the RDoC system in association with suicide (37). Unlike other risk factors, resting-state HR and HRV measures can be monitored on a real-time basis using recent technology (6). With this newly developed technology, clinicians might predict and prevent suicide in a more effective and timely manner. Additional study is needed to apply resting-state HR and HRV to evaluating acute suicide risk on a real-time basis.

The current study findings should be interpreted within the context of the study design. First, this study only included cross-sectional, single, and short-term evaluations. Therefore, we could not evaluate causal relationships. We also did not evaluate whether patients with high risk of suicide in our study actually showed suicidal behavior prospectively. However, as mentioned above, the MINI suicidal score showed good predictive validity in a previous study. Second, we could not control for several confounding factors of resting HRV including physical comorbidities, somatic symptoms, physical activity, current medications, body mass index, sleep profile, timing and smoking status. Third, the study did not include healthy controls.

Notwithstanding the abovementioned limitations, this study demonstrated the associations of resting-state HR and RMSSD with proximal suicide risk in patients with diverse psychiatric conditions. Across diverse psychiatric populations, including those with subthreshold disorder manifestations, we found that resting HR and RMSSD displayed significant associations with proximal suicide risk. In addition, we found that resting HR and RMSSD demonstrated significant associations with proximal suicide risk among patients with any suicide-related symptom (MINI suicidal score >0). Additional research with real time monitoring is needed to confirm the study findings.

## Data Availability Statement

The original contributions presented in the study are included in the article/Supplementary Material, further inquiries can be directed to the corresponding author/s.

## Ethics Statement

The studies involving human participants were reviewed and approved by Samsung medical center. Written informed consent for participation was not required for this study in accordance with the national legislation and the institutional requirements.

## Author Contributions

JB developed the initial research idea. JB, DL, and YC conducted statistical analyses. JB and DL wrote the manuscript. DL and YC constructed the table and figures. JB, DL, YC, and KH edited the finalized version. All authors contributed to the article and approved the submitted version.

## Conflict of Interest

The authors declare that the research was conducted in the absence of any commercial or financial relationships that could be construed as a potential conflict of interest.
