# Supplementary material for: Association of Resting Heart Rate and Heart Rate Variability With Proximal Suicidal Risk in Patients With Diverse Psychiatric Diagnoses
Source: Front Psychiatry. 2021 Apr 30;12:652340. doi: 10.3389/fpsyt.2021.652340 (PMC8121144; doi:10.3389/fpsyt.2021.652340)
Supplement: Supplementary file 1 [file Data_Sheet_1.docx]

**Supplementary table 1 |** Multivariate linear regression analysis of association of resting heart rate (HR) and root-mean-square of R-R interval successive difference with the MINI suicidal scale including participants who reported any kind of suicide-related symptom (MINI suicidal score>0)

| **Model 1: HR as an independent variable** | | | | | |
| --- | --- | --- | --- | --- | --- |
|  | **Unstandardized beta** | **SE** | **Standardized beta** | **t** | ***P*** |
| HR | 0.068 | 0.027 | 0.112 | 2.570 | 0.010 |
| Age | -0.040 | 0.026 | -0.073 | -1.570 | 0.117 |
| Sex | 1.055 | 0.708 | 0.066 | 1.490 | 0.137 |
| HAM-D score | 0.319 | 0.080 | 0.264 | 3.994 | <0.001 |
| HAM-A score | -0.051 | 0.071 | -0.047 | -0.719 | 0.473 |
| Primary diagnosis, MDD | 3.113 | 0.762 | 0.203 | 4.084 | <0.001 |
| Primary diagnosis, bipolar disorder | 4.877 | 1.111 | 0.217 | 4.388 | <0.001 |
|  |  |  |  |  |  |
| **Model2: RMSSD as an independent variable** | | | | | |
|  | **Unstandardized beta** | **SE** | **Standardized beta** | **t** | ***P*** |
| RMSSD | -0.091 | 0.029 | -0.142 | -3.098 | 0.002 |
| Age | -0.081 | 0.027 | -0.148 | -3.044 | 0.002 |
| Sex | 0.868 | 0.710 | 0.054 | 1.223 | 0.222 |
| HAM-D score | 0.331 | 0.079 | 0.274 | 4.171 | <0.001 |
| HAM-A score | -0.062 | 0.071 | -0.057 | -0.873 | 0.383 |
| Primary diagnosis, major depressive disorder | 3.091 | 0.760 | 0.201 | 4.067 | <0.001 |
| Primary diagnosis, bipolar disorder | 4.901 | 1.108 | 0.218 | 4.424 | <0.001 |

*MINI, Mini-International Neuropsychiatric Interview; HR, resting state heart rate; SE, standard error; HAM-D, Hamilton depression scale; HAM-A,Hamilton anxiety scale; RMSSD, root mean square of R-R interval of successive differences.*

**Supplementary table 2 |** Multivariate logistic regression analysis of association of resting heart rate (HR) and root-mean-square of R-R interval successive difference with the moderate-to-high suicide risk including participants who reported any kind of suicide-related symptom (MINI suicidal score>0)

| **Model 1: HR as an independent variable** | | | |
| --- | --- | --- | --- |
|  | **aOR** | **95% CI** | ***P*** |
| HR | 1.021 | 1.004-1.038 | 0.017 |
| Age | 0.983 | 0.968-0.999 | 0.039 |
| Sex | 0.812 | 0.525-1.255 | 0.348 |
| HAM-D score | 1.152 | 1.093-1.215 | <0.001 |
| HAM-A score | 0.955 | 0.913-0.998 | 0.042 |
| Primary diagnosis |  |  | <0.001 |
| - Major depressive disorder | 2.684 | 1.654-4.355 | <0.001 |
| - Bipolar and related disorder | 3.431 | 1.862-7.475 | <0.001 |
| **Model 2: RMSSD as an independent variable** | | | |
|  | **aOR** | **95% CI** | ***P*** |
| RMSSD | 0.977 | 0.959-0.995 | 0.012 |
| Age | 0.972 | 0.955-0.989 | <0.001 |
| Sex | 0.846 | 0.546-1.311 | 0.454 |
| HAM-D score | 1.156 | 1.097-1.219 | <0.001 |
| HAM-A score | 0.952 | 0.911-0.996 | 0.032 |
| Primary diagnosis |  |  | <0.001 |
| - Major depressive disorder | 2.684 | 1.655-4.353 | <0.001 |
| - Bipolar and related disorder | 3.770 | 1.878-7.566 | <0.001 |

*MINI, Mini-International Neuropsychiatric Interview; HR, resting state heart rate; SE, standard error; HAM-D, Hamilton depression scale; HAM-A,Hamilton anxiety scale; RMSSD, root mean square of R-R interval of successive differences*
